# Supplementary material for: The interactome of intact mitochondria by cross-linking mass spectrometry provides evidence for coexisting respiratory supercomplexes
Source: Mol Cell Proteomics. 2017 Dec 8;17(2):216–32. doi: 10.1074/mcp.RA117.000470 (PMC5795388; doi:10.1074/mcp.RA117.000470)
Supplement: Supplemental Data [file supp_RA117.000470_133922_0_supp_23978_fzffwf.zip › spectra_annotation/mito_spectra_annotation/474-1-5-1-128-1.pdf]

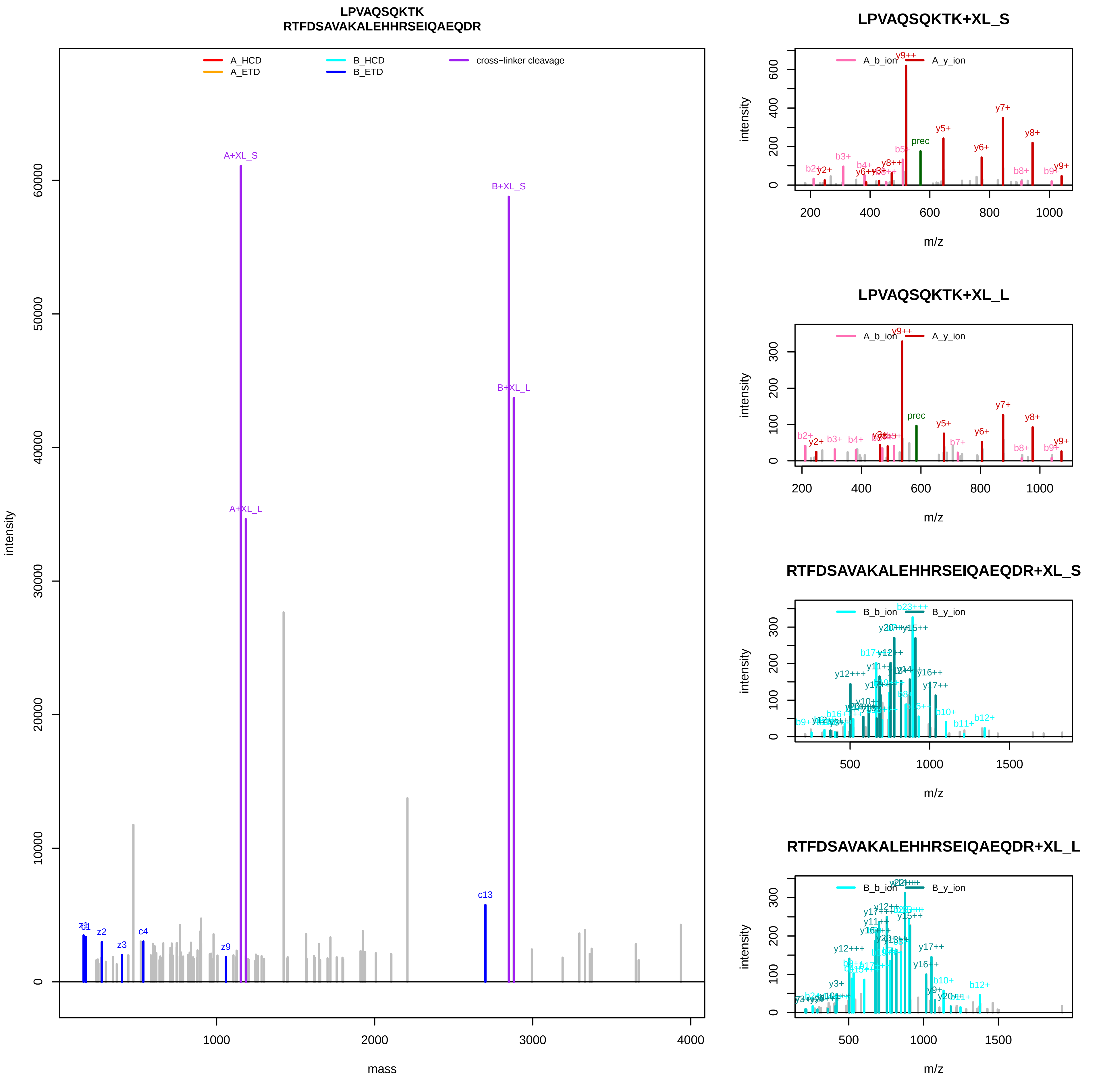

LPVAQSQKTK
RTFDSAVAKALEHHRSEIQAEQDR
A_HCD
A_ETD
B_HCD
B_ETD
cross−linker cleavage
A+XL_S
B+XL_S
B+XL_L
A+XL_L
c13
z1
c1
z2
z3
c4
z9
intensity
mass
0
10000
20000
30000
40000
50000
60000
1000
2000
3000
4000
LPVAQSQKTK+XL_S
LPVAQSQKTK+XL_L
RTFDSAVAKALEHHRSEIQAEQDR+XL_S
RTFDSAVAKALEHHRSEIQAEQDR+XL_L
A_b_ion
A_y_ion
B_b_ion
B_y_ion
prec
y9++
y7+
y5+
y6+
y8+
y9+
b2+
y2+
b3+
b4+
b5+
b7+
b8+
b9+
b10+
b11+
b12+
y12+++
y16++
y17++
y15++
y3+
m/z
200
400
600
800
1000
500
1500
